# *LIA5* Is Required for Nuclear Reorganization and Programmed DNA Rearrangements Occurring during *Tetrahymena* Macronuclear Differentiation

**DOI:** 10.1371/journal.pone.0075337

**Published:** 2013-09-17

**Authors:** Annie Wan Yi Shieh, Douglas L. Chalker

**Affiliations:** Biology Department, Washington University in St. Louis, St. Louis, Missouri, United States of America; St Jude Children's Research Hospital, United States of America

## Abstract

During macronuclear differentiation of the ciliate *Tetrahymena thermophila*, genome-wide DNA rearrangements eliminate nearly 50 Mbp of germline derived DNA, creating a streamlined somatic genome. The transposon-like and other repetitive sequences to be eliminated are identified using a piRNA pathway and packaged as heterochromatin prior to their removal. In this study, we show that *LIA5*, which encodes a zinc-finger protein likely of transposon origin, is required for both chromosome fragmentation and DNA elimination events. Lia5p acts after the establishment of RNAi-directed heterochromatin modifications, but prior to the excision of the modified sequences. In *∆LIA5* cells, DNA elimination foci, large nuclear sub-structures containing the sequences to be eliminated and the essential chromodomain protein Pdd1p, do not form. Lia5p, unlike Pdd1p, is not stably associated with these structures, but is required for their formation. In the absence of Lia5p, we could recover foci formation by ectopically inducing DNA damage by UV treatment. Foci in both wild-type or UV-treated *∆LIA5* cells contain dephosphorylated Pdd1p. These studies of *LIA5* reveal that DNA elimination foci form after the excision of germ-line limited sequences occurs and indicate that Pdd1p reorganization is likely mediated through a DNA damage response.

## Introduction

DNA damage threatens genome integrity and must be efficiently repaired to prevent mutations or aberrant chromosomal rearrangements. DNA double-strand breaks (DSB) are among the most deleterious DNA lesions. They occur frequently, either as a consequence of environmental insults or stress on DNA introduced by essential cellular processes, including transcription and DNA replication. DSB are also introduced as part of intrinsic cellular programs. Spo11 induced breaks trigger homologous recombination during meiosis, and the Rag1/2 recombinase initiates immunoglobulin gene rearrangement during vertebrate lymphocyte maturation [1,2].

Given their prevalence and severity if left unattended, it is not surprising that cells have multiple means to mend these lesions. DSBs are repaired by two major pathways – Homologous Recombination (HR) and Non-Homologous End Joining (NHEJ) (see 3). HR is used primarily when an undamaged donor strand is available to template repair (e.g. repair of stalled replication forks). The Rad51 protein is a major player in this pathway, binding to single-stranded DNA after exonucleolytic processing of the damage DNA. NHEJ is the major pathway for repairing non-replication associated breaks. Catalysis of NHEJ repair involves the binding of broken ends by the Ku70/Ku80 heterodimer, which results in the recruitment and activation of the DNAPK complex. After processing, the broken ends are rejoined by DNA ligase IV in association with its partner XRCC4.

Upon sensing lesions in DNA, cells respond by transducing a cascade of signals to induce repair. This is collectively referred to as the DNA damage response (DDR). This process activates effector proteins that ensures proper amplification and transmission of the repair signal to facilitate repair, as well as evokes cellular responses to either stall damaged cells in their cell cycle or trigger apoptosis in cells that fail to resolve their DNA breaks. Activation of the DDR is evident by the phosphorylation of Histone variant H2AX (γH2AX) and the formation of DNA repair foci [4-7]. DNA repair foci represent the ordered assembly of repair factors at the sites of the lesions to effect the healing of the damage DNA (reviewed in 8). These events suggest that extensive chromatin remodeling occurs upon DNA damage.

DNA repair is influenced by nuclear architecture (see 8). Evidence suggest that the repair of DSB occurs with slower kinetics in heterochromatin compared to euchromatin [9]. As heterochromatin domains are rich in repetitive sequences, it is necessary to carefully regulate repair to prevent genomic instability. Improper recombination between distal homologous sequences may lead to deleterious inversions or translocations of chromosomal sequences. A major heterochromatin component, heterochromatin protein 1 (HP1) has been shown to play a critical role in DNA damage repair (reviewed in 10-12). While some studies suggest that HP1 mobilization facilitates repair by allowing accessibility of repair machineries to damage sites, a more direct role for the actual process of repair have been implicated. In fact, it has been suggested that HP1 acts as an important target of the DDR.

The programmed genome rearrangements of the ciliate *Tetrahymena thermophila* provide an opportunity to examine the interplay between heterochromatin and DNA repair. During *Tetrahymena* somatic nuclear differentiation, nearly 50 Mb of germline-derived DNA are packaged as heterochromatin and eliminated by site-specific recombination (reviewed in 13,14). *Tetrahymena* are single cell eukaryotes that exhibit nuclear dimorphism, where two morphologically distinct nuclei contain different copies of the genome that individually act as the germline and the soma [15]. The germline micronucleus houses a diploid genome that is transcriptionally silent during vegetative growth, divides mitotically, and exists to maintain and transmit genetic information to sexual progeny. Conversely, the somatic macronucleus is responsible for all gene expression necessary to support growth. Its genome is polyploid and highly fragmented. The macronucleus is a terminally differentiated nucleus, which divides amitotically, and is lost during sexual reproduction when a new macronucleus is formed from the parental germline.

During sexual reproduction, future micro- and macronuclei receive copies of a common zygotic genome, formed by kayrogamy after cross-fertilization with meiotic products derived from mating partners’ germline micronuclei (reviewed in 16). As macronuclei differentiate, 5000-6000 dispersed loci are identified and targeted for elimination [17-19]. In addition the germline-derived chromosomes undergo chromosome breakage (at ~180 sites) coupled with *de novo* telomere addition [20-22]. These DNA rearrangements remove germline-limited transposons and many non-coding sequences, which are collectively called Internal Eliminated Sequences (IESs). The recognition of IESs occurs through small RNA directed heterochromatin formation [23,24]. The small RNAs are generated during meiosis, early into *Tetrahymena* conjugation, by processing micronuclear, bi-directional transcripts into ~28nt scan (scn) RNAs by the Dicer Like 1(Dcl1) protein [23,25,26]. Later in the early differentiating macronuclei, the scnRNAs homologous to IESs target their complementary loci for the deposition of Histone H3 lysine (K) 9 and K27 methylation [24,27]. These marks recruit chromodomain-containing proteins Pdd1p and Pdd3p [28,29]. This newly established IES heterochromatin then assembles with additional factors that lead to the generation of IES–containing nuclear foci [30]. Whether these so-called DNA elimination foci form before, after, or concurrent with IES excision is an unanswered question.

The targeting of heterochromatin modifications to IESs during *Tetrahymena* nuclear reprogramming has clear mechanistic similarities to the way that the metazoan piRNA pathway silences transposable elements (see 13). Many IESs are likely derived from ancestral transposons and new invading elements in the genome. In fact, it appears that ciliates have co-opted transposon proteins to carry out the elimination of small RNA-directed heterochromatin. In both *Paramecium* and *Tetrahymena*, a domesticated *piggyBac* transposase serves as the excisase that cuts IESs out of the developing somatic genome [31,32]. NHEJ proteins then assist in healing these developmentally regulated DNA breaks as the DNA LigaseIV/XRCC4 complex and Ku80 have been shown to be essential for DNA rearrangement in *Paramecium* and *Tetrahymena*, respectively [33,34]. Thus much like the process of V(D)J recombination, which uses a domesticated transposase – RAG1/2, ciliate DNA rearrangements appear to have evolved by recruiting a transposase protein to perform developmentally programmed DNA repair.

In this study, we investigated the role of a Lia5p, a developmentally expressed nuclear protein, in programmed DNA rearrangement [30]. We found that Lia5p is required for IES excision, acting after the establishment of heterochromatin in developing macronuclei. In *∆LIA5* cells, DNA elimination foci do not form, which further reveals defects in DNA rearrangement; however foci formation could be induced by ectopically introducing DNA damage. In both wild-type foci and UV-induced foci, Pdd1p is dephosphorylated, an observation that, together with other data, reveals that foci form after and in response to programmed DNA breaks.

## Results

### Lia5p, a transposon-related protein is essential for development

**Figure 1:**
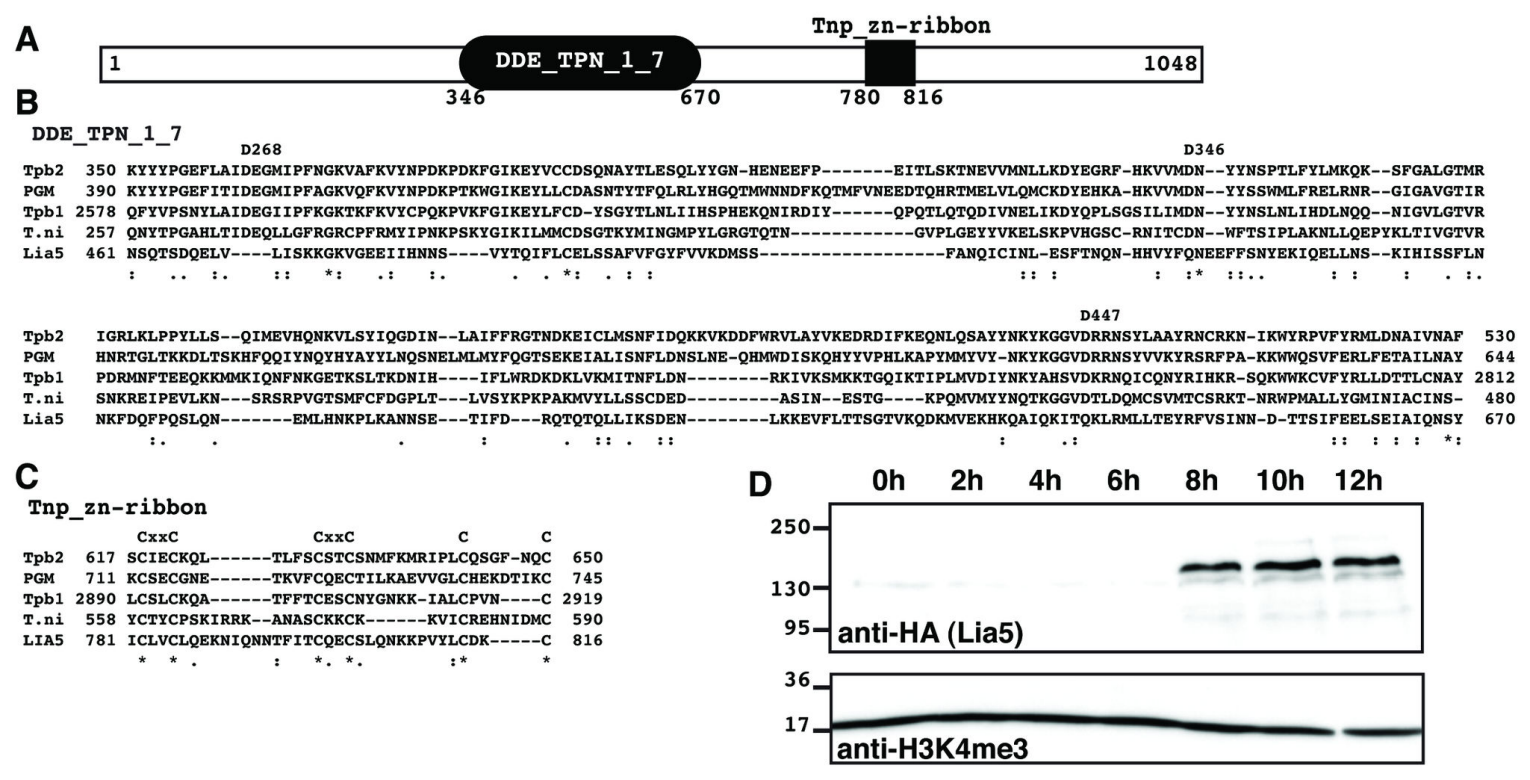
Lia5p is a developmentally expressed transposon-like protein. (A) Representation of Lia5p showing the positions of conserved DDE_Tnp_1_7 and domains. Alignment of Lia5p with (B) the DDD/E and (C) Tnp_zfribbon_2 domains of *Trichoplusia ni* *piggyBac* transposon and ciliate domesticated transposases, *Paramecium* PGM and *Tetrahymena* Tpb2p and Tpb1p: the *T. ni* catalytic core residues are noted above the alignment. (D) Western blot analyses showing Lia5 mRNA and protein expression from 0 to 12 hrs of conjugation; the hour of conjugation is indicated above each lane. Detection of the abundant histone H3 tri-methylated on K4.

Previously, we identified several LIA (Localized In macronuclear Anlagen) genes encoding proteins that were expressed exclusively during macronuclear differentiation and showed that *LIA1* was required for programmed genome rearrangements [30,35]. Most Lia proteins (p), including Lia1p had no clear orthologs and few conserved domains making the prediction of function challenging. *LIA5* was initially described as encoding a 1048 amino acid (aa) glutamine-rich protein containing a putative FVYE or PHD-type zinc finger, but more recent analyses have revealed that this motif shows similarity to a zinc ribbon domain (pfam13842: Tnp_zf-ribbon_2) commonly found at the C-terminus of transposon-derived proteins (Figure 1A, C). Furthermore, the central region of Lia5p shares similarity with the IS4 transposase family (pfam13843: DDE_Tnp_1_7) [36], which includes the *Tetrahymena piggyBac* transposase (Tbp2p) [32] (Figure 1A, B). Despite this structural similarity, alignment of Lia5p to Tpb2p and other predicted transposases showed that Lia5p apparently lacks the DDD catalytic triad found in active transposases (Figure 1B). Domestication of transposon-derived proteins, including Rag1/2 and Tbp2p, has created novel pathways acting on eukaryotic chromosomes. Our data suggest that Lia5p may have similarly evolved from domestication of a transposable element protein.

We previously found that *LIA5* mRNA accumulates rapidly between 6 and 9 hours after initiation of conjugation before its levels drop significantly by 12 hours [30]. To determine how Lia5p accumulates relative to the mRNA, we tagged the endogenous gene on its amino terminus with a hemagglutinin (HA) epitope and examined its expression. HA-Lia5p was first detected at 8 hrs into conjugation indicating that protein expression is delayed until new macronuclei form (Figure 1D). Unlike mRNA expression, we observed no detectable decrease in protein levels at 12 hours. This is consistent with our previous observation that GFP-Lia5p localizes to the developing macronuclei throughout differentiation [30].

**Figure 2:**
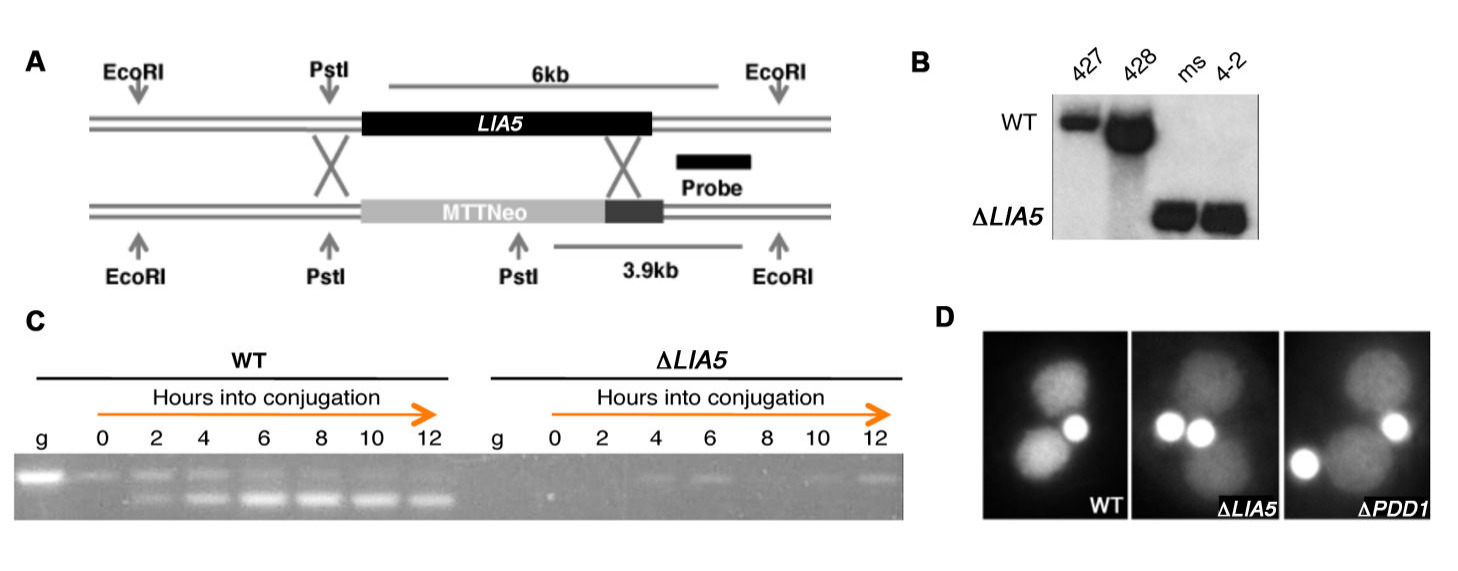
*LIA5* is essential to complete conjugation. (A) Illustration of LIA5 gene replacement with the NEO3 selectable marker (MTTNeo), which confers paromomycin resistances upon induction with CdCl2. “X″ s denote homologous recombination directing gene replacement. Arrows indicate restriction enzyme cut sites used to verify gene disruption by Southern blot analysis (B). The region labeled for use as a probe is shown as a black bar. It detects a 6kb (WT) or a 3.9kb (Δ*LIA5*) EcoRI-PstI fragment indicative of the wt *LIA5* or knockout allele, respectively. DNA was isolated from wild type (CU427, CU428) and ∆*LIA5* strains (ms, 4-2). (C) rtPCR for the expression of *LIA5* in conjugating wild type (WT) and *LIA5* knockout (Δ*LIA5*) at indicated hours into conjugation. Primers used span the sixth intron and detect a smaller mRNA product that is easily distinguished from the product amplified from genomic DNA (g) used as control for amplification (or background from possible minor DNA contamination in the PCR). (D) Fluorescent images of representative DAPI stained WT, *∆LIA5* and *∆PDD1* strains post conjugation.

To determine whether Lia5p is essential for macronuclear differentiation, we deleted all copies of *LIA5* from both the micro- and macronuclear genome by homologous gene replacement with the *neo3* paramomycin-resistance cassette [37] (Figure 2A). The absence of the gene in *LIA5* knockout (Δ*LIA5*) cell lines was verified by Southern blot analysis, and the loss of all expression was confirmed using rtPCR (Figure 2B, C). When Δ*LIA5* strains were mated, no viable progeny were produced indicating that this gene has an essential role during conjugation.

To determine the stage of development that knockout cells failed to complete, we examined the nuclear morphology of the cells throughout conjugation. Δ*LIA5* cells progressed through early stages (meiosis, nuclear exchange and karyogamy, and formation of new macronuclei) at a rate that was similar to that of wild type (wt) cells with no obvious developmental delays (data not shown). WT mating cells complete conjugation after pair separation by eliminating one of two micronuclei, producing cells that have two newly differentiated macronuclei and one micronucleus. These cells are poised to divide the one remaining micronucleus and undergo cytokinesis once fed. We found that Δ*LIA5* cells arrested as exconjugants, prior to elimination of one micronucleus. Furthermore, the new macronuclei within mutant conjugants failed to fully amplify their genomic DNA as indicated by the weak intensity of DAPI staining relative to wt. This two macronuclei/two micronuclei terminal arrest phenotype has been commonly observed in mutants lacking genes (such as *DCL1, PDD1* and *LIA1*) that are required for programmed DNA rearrangements [25,26,35,38].

### *LIA5* is required for DNA elimination and chromosome fragmentation

**Figure 3:**
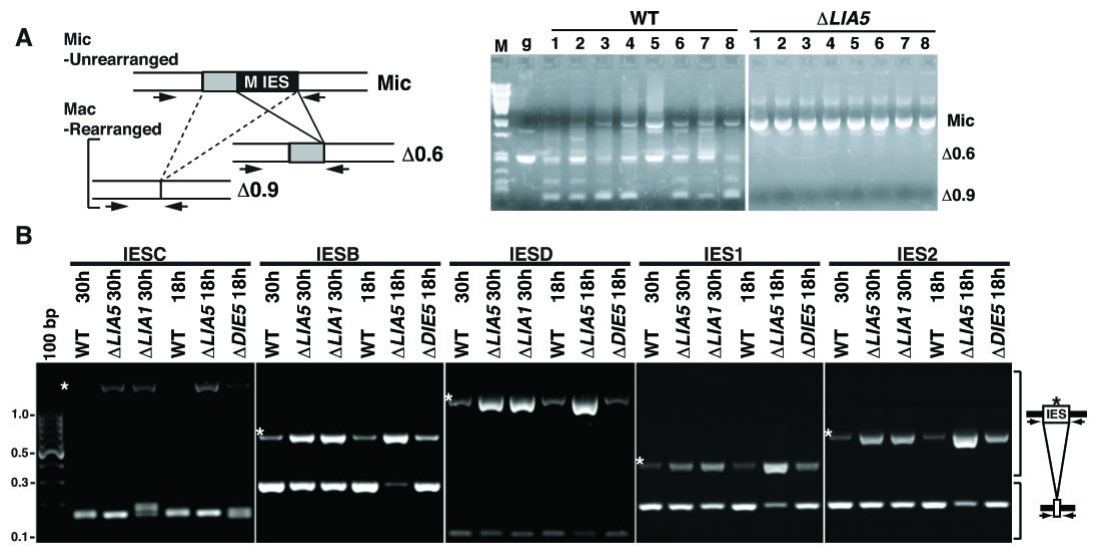
*LIA5* is required for Tetrahymena programmed DNA rearrangement. (A) The illustration shows the unrearranged M IES (Mic) and the two major alternative rearrangement that excise either 0.6kbp (Δ0.6) or 0.9kbp (Δ0.9). Arrows denote forward and reverse primers used in the second round of nested PCR to amplify across the M IES locus. PCR of single cells to assess M IES excision; M, PstI-digested Lambda DNA size marker; g, genomic DNA from unmated CU428 cells. Each lane (1-8) represents a single exconjugant from WT or *∆LIA5* crosses. The expected positions for the unrearranged (Mic) and rearranged (Δ0.6 and Δ0.9) products are indicated to the right. (B) Genomic DNA isolated from WT or mutant strains 18 or 30 hours after initiating conjugation (as indicated) was amplified by PCR using primers (designated by arrowheads) flanking five different IESs site. PCR products from IES-containing or IES-eliminated DNA regions are indicated with brackets. The size of relevant DNA standards is indicated at left.

Because the developmental arrest of Δ*LIA5* cells has been commonly observed for mutants that fail to complete DNA rearrangements, we examined loci that undergo either DNA elimination or chromosome breakage to determine whether *LIA5* is required for these somatic genome-remodeling events. The M IES is a well-characterized eliminated sequence (Figure 3A). We isolated single exconjugants from either wt or Δ*LIA5* mating populations and used nested PCR to assess this element’s rearrangement status. Each successful rearrangement of this IES generates one of two alternative products, removing either 0.6kb or a 0.9kb [39]. PCR using primers flanking the IES can detect both of these rearranged products as well as any unrearranged loci. M IES rearrangement was readily detected in wt exconjugants as the predominant PCR products were less that 600 bp. In contrast, in Δ*LIA5* mated cells we detected accumulation of a larger product migrating at the size expected for the unrearranged, germ line form (Figure 3B). We also isolated DNA from wt or ∆*LIA5* populations post-conjugation and examined the rearrangement of the L,M,R locus, which contains two IESs in addition to M. Southern blot analysis showed that the all three IESs failed to be excise in cell lacking *LIA5* (data not shown).

Thousands of IESs are dispersed throughout the germline-derived genome of each differentiating macronucleus. To assess whether *LIA5* is necessary of excision of many of these, we selected five recently identified IESs in a genome-wide mapping study [19]. Genomic DNA isolated from wt or mutant exconjugants (either 18 or 30 hours after mixing strains to initiate mating) was used for template in PCR with primer pairs flanking each IES. The unrearranged form of each IES is easily detectable in ∆*LIA5* and ∆*LIA1* cells [35] relative to wt showing that without *LIA5*, cells are unable to excise these IESs from their developing macronuclei. The detection of unrearranged IESs in ∆*LIA5* cells was comparable to that observed in ∆*LIA1* cells, but was in marked contrast to what we observed at these loci in ∆*DIE5* cells (Figure 3B). We previously showed that rearrangement of the M IES locus required zygotic expression of *DIE5* by using an assay that detects joining of the macronuclear retained DNA after excision. In Figure 3B, ∆*LIA5* cells clearly accumulated unrearranged DNA, but ∆*DIE5* cells did not. This difference is consistent with failure of ∆*LIA5* cells to initiate breaks, whereas ∆*DIE5* cells initiate, but cannot repair breaks; this leads *DIE5* mutants to degrade their macronuclear DNA [40].

**Figure 4:**
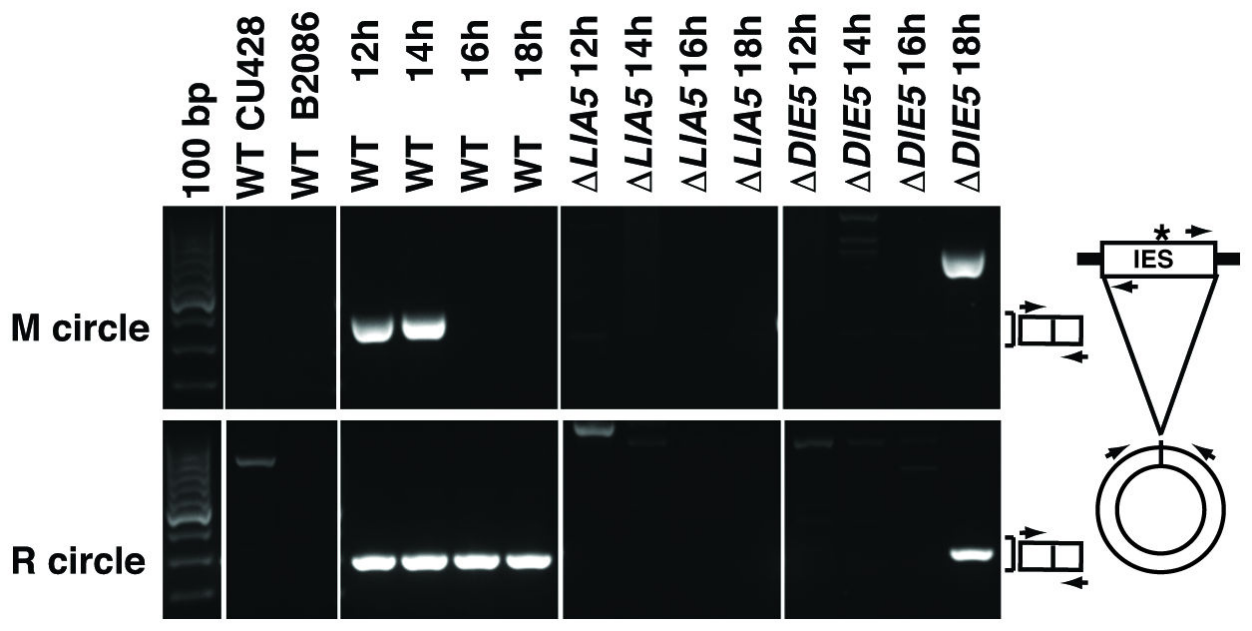
Δ*LIA5* fails to excise IES. Genomic DNA was isolated from mating WT, *∆LIA5*, or *∆DIE5* cells between 12 and 18 hours after initiating conjugation and used as template in nested PCR reactions to detect excised circles from the M or R IES. The position of round 2 primers (arrowheads) is schematized on the right and the expected size for properly excised IES circle products is indicated by the brackets. A 100 bp ladder was used as a size standard.

Excision of IESs from the developing genome produces circular forms that can be detected by inverse PCR [33,41]. To further assess whether or not ∆*LIA5* cells can initiate IES excision, we isolated genomic DNA between 12 and 18 hours of conjugation and tested for IES circle generation in wt and mutant cells. Circular forms of both the M and R IESs were easily detectable in multiple time points in wt cells, but were never observed in ∆*LIA5* cells for any time point from two different mating populations (Figure 4 and data not shown). Circular forms of IES were observed in ∆*DIE5* cells, albeit at decreased frequency than they were detected in wt cells. This is consistent with our interpretation that IES excision occurs in the absence of Die5p, but a failure in downstream joining events leads to loss of macronuclear DNA.

**Figure 5:**
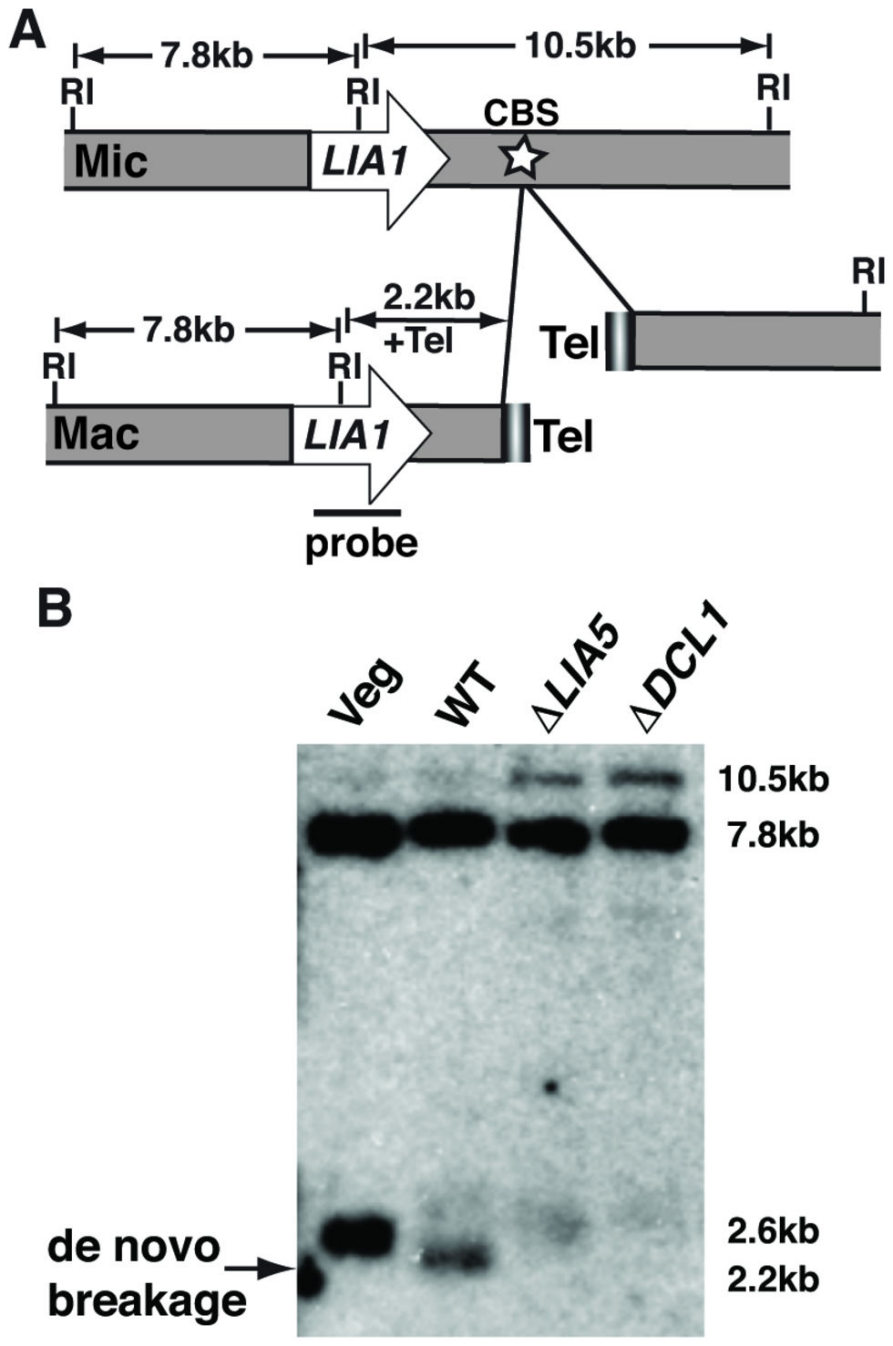
*LIA5* is required for chromosome breakage. (A) The diagram illustrates the *LIA1* locus and flanking DNA showing the downstream chromosomal breakage sequence (CBS) (star symbol). *Eco*RI (RI) restriction sites used for the Southern blot analysis are shown. The probe spans the central *Eco*RI site and detects: 1) a 7.8 kbp distal fragment common to both micro- and macronuclei; 2) a 10.5kbp micronucleus-specific fragment; 3) a 2.2kbp fragment indicating *de* *novo* breakage; and 4) variable 2.5kb to 2.6kbp fragments indicative of parental macronuclear chromosomes with fully elongated telomeres (Tel), remaining due to the unmated cells in the population. (B) Southern blot analysis of *Eco*RI digested genomic DNA from unmated (Veg) or mated populations of WT or mutant strains was used to assess chromosome breakage. The arrow indicates the 2.2 kbp fragment consistent with *de* *novo* breakage; the approximate sizes of the other fragments are indicated to the right.

In addition to IES excision, *Tetrahymena* macronuclear differentiation involves breakage of chromosomes at ~180 loci followed by *de novo* telomere addition. Although the connection between chromosome breakage and IES excision is poorly understood, strains lacking genes that are required for IES excision frequently fail to fragment chromosomes as well. To test whether *LIA5* is required for this process, we examined the chromosome breakage site found just downstream of the *LIA1* gene [25]. DNA isolated from post-conjugation populations of wt or mutant cells was digested with EcoRI and analyzed by Southern blot using a *LIA1*-specific radiolabeled probe (Figure 5A). In wt exconjugant populations, *de novo* breakage is readily observed as a ~2.2kb fragment, which migrates faster than the mature macronuclear form (at ~2.5-2.6kb), which has fully elongated telomeres (the major form detected in vegetatively growing cells and observed in the post-conjugative populations due to unmated cells in the populations tested) (Figure 5B). No chromosome breakage is observed in ∆*LIA5* or ∆*DCL1* mutant populations, evident both by the absence of the 2.2kb fragment and increased abundance of the unrearranged micronuclear form migrating at 10.5kb. Thus *LIA5* is required for both IES excision and chromosome breakage.

### Δ*LIA5* strains establish heterochromatin, but fail to reorganize it into nuclear foci

**Figure 6:**
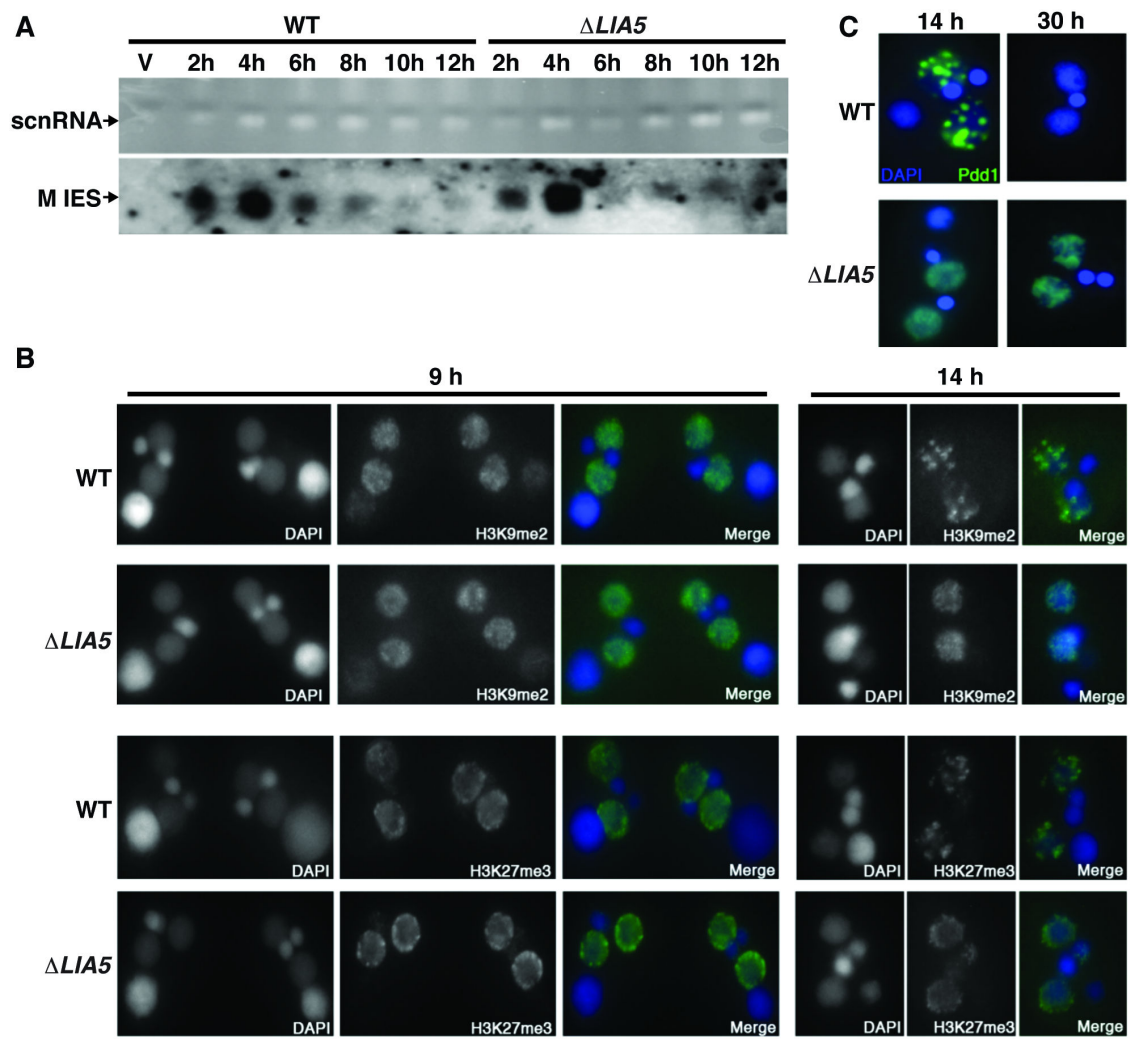
Lack of *LIA5* causes failure in IES excision foci formation while critical events prior to the step remain unperturbed. (A) ScnRNAs accumulate to wt levels: upper panel: ethidium bromide staining of total RNA (20 µg per lane) extracted from starved (s) or conjugating WT or *∆LIA5* cells at the indicated time points (2-12hrs). Lower panel: Northern blot analysis using an M IES-specific probe. (B) Immunofluorescence staining of histone H3K27me3 and H3K9me2 levels in WT and *∆LIA5* at 9hrs and 14hrs into conjugation. (C) Fluorescent images of Pdd1-YFP localization in the developing macronuclei of WT and *∆LIA5* conjugating cells, counterstained with DAPI. The 30hr time point represents mated cells that have completed development or arrested prior to the wt end-point.

IESs are targeted for elimination from developing macronuclei through small RNA directed heterochromatin formation [23,24,27]. To determine whether Lia5p acts in the establishment of heterochromatin modifications or in downstream events, we examined the ability of ∆*LIA5* cells to complete the critical steps in the macronuclear development. Wt cells generated germline specific scnRNAs during meiosis that direct histone H3 K9 and K27 methylation to homologous IES in differentiating macronuclei (Figure 6). Mating populations of ∆*LIA5* accumulated wt levels of scnRNAs (Figure 6A) and acquired methylation (me) on K9(me2,3) and K27(me3) of histone H3 (Figure 6B, 9hrs). These findings were not unexpected as the peak of Lia5p expression occurs after these events.

Upon establishment of heterochromatin modifications on IES, proteins required for IES excision, including the chromodomain-containing protein Pdd1p, assemble on the modified chromatin, which is followed by the redistribution of modified chromatin into nuclear foci (Figure 6B, WT-14hrs). The purpose of organizing IESs into these DNA elimination foci is not known, but it has been suggested that their formation facilitates IES excision and/or the degradation of the associated germline-limited DNA. Even though ∆*LIA5* cells establish heterochromatin modifications, the nuclear reorganization of the modified sequences does not occur as both H3K9me2 and K27me3 remains dispersed throughout the developing somatic macronuclei (Figure 6B, ∆*LIA5*-14hrs). The failure of nuclear reorganization is not due to obvious deficiencies of Pdd1p production. In ∆*LIA5* cells, Pdd1p accumulates to levels indistinguishable from levels observed in wild-type cells by 9hrs (Figure S1). In fact, both Pdd1p and Pdd3p levels remain at peak levels in ∆*LIA5* cells until at least 15 hours, a time point when these proteins would normally be eliminated along with IES chromatin.

Clearly, partitioning thousands of loci into a countable number of distinct foci necessitates massive nuclear reorganization, which is readily visualized through tracking the dynamic localization of the essential chromodomain protein Pdd1p [28,30,42]. Pdd1p, like the methylated IES chromatin to which it binds, is initially dispersed throughout the developing macronucleus, then assembles into condensed nuclear foci coincident with the onset of IES excision. To examine how loss of *LIA5* affects nuclear reorganization, we followed the localization of Pdd1p tagged with yellow fluorescent protein (YFP). Whereas Pdd1-YFP localized to distinct nuclear foci in wt exconjugants (Figure 6C, WT-14hrs), it remained dispersed in the developing macronuclei of ∆*LIA5* cells, even 30 hrs after initiating mating when the fusion protein had disappeared from the fully differentiated macronuclei of wt cells, presumably as IESs were eliminated (Figure 6C, 30hrs).

**Figure 7:**
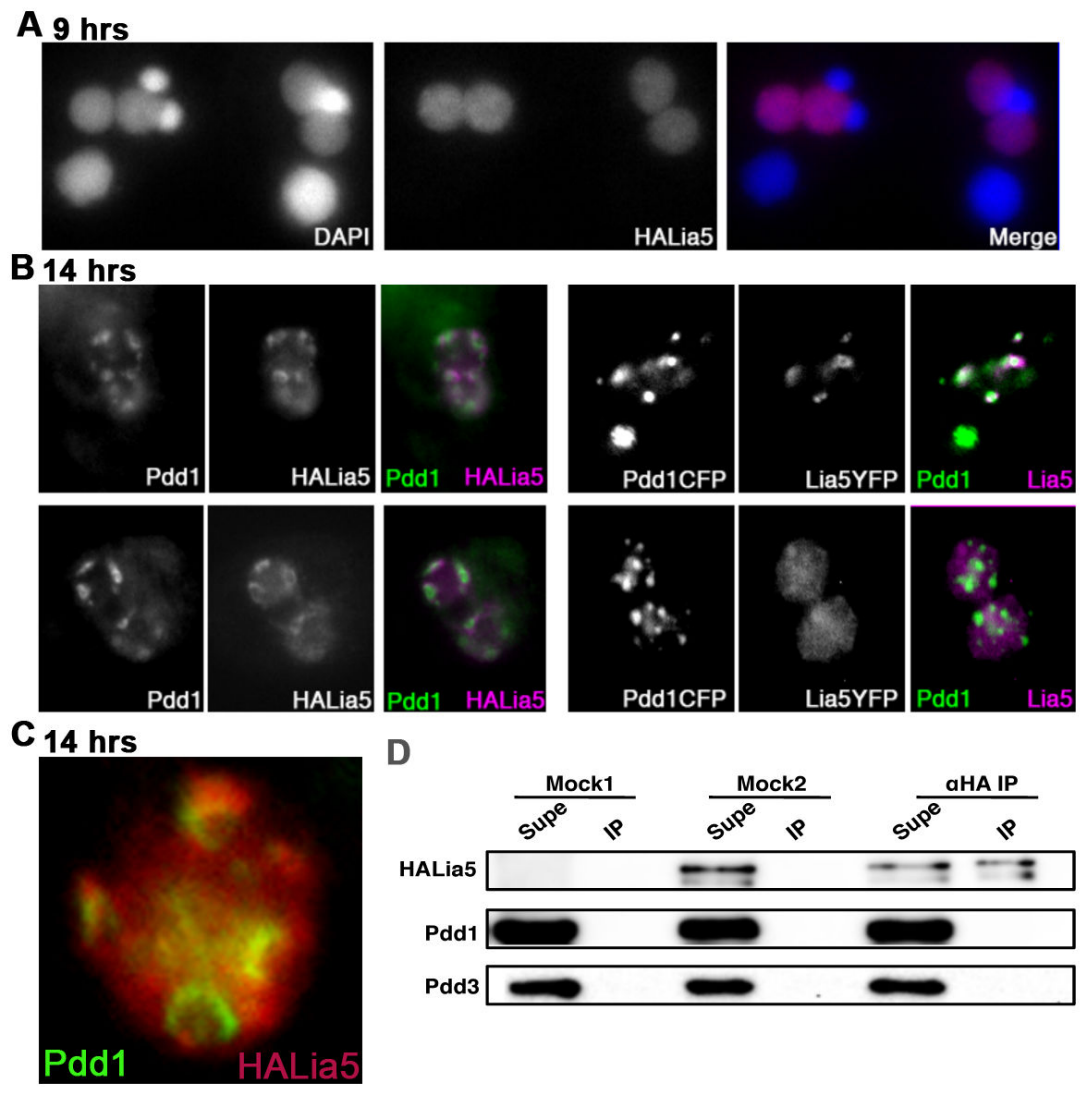
Lia5 localizes to DNA elimination foci, but not within the Pdd1 central core. (A) Immunostaining of HALia5 at 9hrs into conjugation. (B, C) Co-localization of Lia5-YFP and Pdd1-CFP or immuno-stained HALia5 and Pdd1-YFP in 14hrs conjugating cells. In (C), Pdd1p and Lia5 localization are shown together in a single developing macronucleus. (D) Immunoprecipitation of HALia5 (anti-HA IP). Untransformed cells (Mock1) and HALia5 transformant lysates immunoprecipitated with rabbit-IgG only (Mock2) are shown as controls. Immunoprecipitated samples (IP) and their respective supernatants (Supe) were analyzed by SDS-PAGE and western blot analysis. HALia5 was visualized with HA epitope antibodies and Pdd1 and Pdd3 were detected specific polyclonal antibodies. HA-Lia5p appears as a doublet after immunoprecipitation, which we have not further investigated.

The above data indicate that Lia5p plays a critical role in the organization of modified IES chromatin into DNA elimination foci. We previously showed that Lia5p and Pdd1p can be found together in large DNA elimination structures [30]. To more closely examine the association of Lia5p with these foci, we co-expressed Pdd1-CFP with either an endogenous N-terminally tagged HA-Lia5p (see Figure 1D) or Lia5-YFP (a C-terminally tagged allele expressed from a high copy rDNA vector) and asked whether Lia5p assembles into Pdd1p-containing foci. Lia5p was detected in the developing macronucleus as soon as they emerged (Figure 7A). As macronuclei developed, Lia5p became increasingly concentrated at distinct regions within macronuclei, occupying similar nuclear domains as Pdd1p. Even so, Lia5p did not consistently co-localize with Pdd1p. Whereas Pdd1-CFP formed compact foci, the tagged Lia5p appeared to concentrate in regions surrounding the Pdd1p foci. This is most clearly observed in HA-Lia5p expressing cells (Figure 7B). The Lia5-YFP also localized surrounding Pdd1-CFP, but appeared more dispersed than HA-Lia5 in some mating pairs. It is possible that the ratio of dispersed: localized protein is altered by the large tag interfering with some Lia5p action as expression of the Lia5-YFP construct in ∆*LIA5* cells did not efficiently rescue the knockout. Nevertheless, as both the HA-Lia5 and Lia5-YFP localize peripherally to Pdd1p foci, our results argue that Lia5p is not a core structural component of these foci. To assess whether Lia5p is recruited to the periphery of DNA elimination foci by Pdd1p, we asked whether these two proteins co-immunoprecipitated from conjugating cells. Immunoprecipitation of HA-Lia5 did not co-precipitate Pdd1p or Pdd3p, which indicates that Lia5 does not stably interact with these core foci components (Figure 7C).

### *LIA5* is required for proper regulation of Pdd1p phosphorylation during conjugation

**Figure 8:**
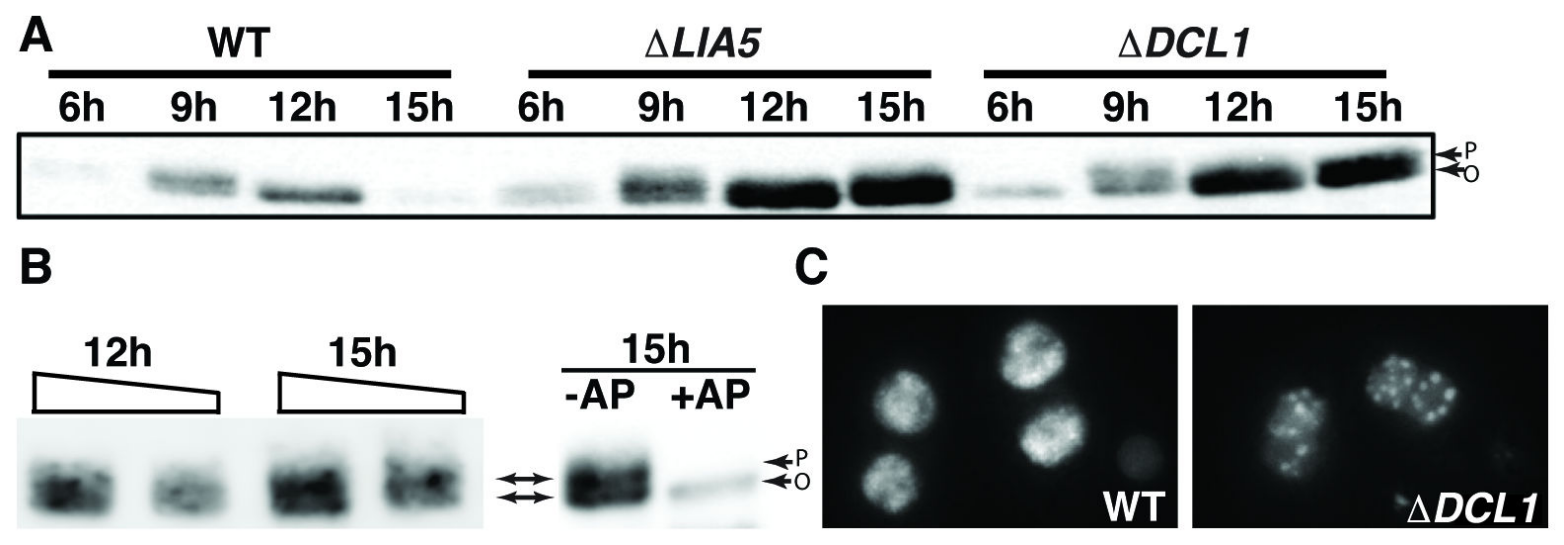
Pdd1 dephosphorylation does not occur in Δ*LIA5* **conjugation** **cells**. (A) anti-Pdd1 western blot analysis of total protein isolated from conjugating WT, *∆LIA5* and *∆DCL1* at indicated time point. (B) Left panel, anti-Pdd1 western blot analysis of two to four fold reduced loading of 12 and 15hrs protein lysates taken from the conjugating Δ*LIA5* samples shown in (A), right panel, Alkaline phosphatase (AP) treatment of the same Δ*LIA5* 15hr lysate. Overall protein levels decrease after treatment due to some proteolytic activity in the lysate. (C) Fluorescent images of premature Pdd1-YFP foci in ΔDCL1 conjugants. P, Phosphorylated Pdd1p; O, unphosphorylated Pdd1p.

Like HP1 proteins of other eukaryotes, Pdd1p shows regulated phosphorylation [28,43], although its importance is largely unexplored. Pdd1p phosphorylation has been shown to peak early during macronuclear differentiation and decrease as macronuclei mature (Figure 8A). Thus, Pdd1p dephosphorylation coincides with foci formation and IES excision. To determine whether dephosphorylation might regulate Pdd1p’s ability to aggregate into foci, we monitored its phosphorylation state in wt and *∆LIA5* mating cells, resolving these forms on 9% SDS polyacrylamide gels, detected with anti-Pdd1p antibodies. For both wt and *∆LIA5*, Pdd1p migrates as a doublet representing the phosphorylated (upper band-P) and unphosphorylated (lower band-O) proteins, which were clearly evident by 9hr. By 12hr when Pdd1p is primarily found in DNA elimination foci, most of the Pdd1p in wt cells appeared to be dephosphorylated as judged by the collapse of the doublet into a single band (Figure 8A). Furthermore, as macronuclear differentiation proceeds, Pdd1p levels decrease, correlating with the period during which IESs are eliminated (wt 12-15hrs). In contrast, Pdd1p continues to accumulate between 12 and 15 hrs in *∆LIA5* mating cells (Figure 8A, S1), which initially obscured the resolution of the Pdd1p doublet (Figure 8A). Underloading of 12hr and 15hr protein samples isolated from *∆LIA5* cells allowed us to see that Pdd1p remained phosphorylated (Figure 8B left panel). Alkaline phosphatase treatment of these samples resulted in collapse of the double to a single band, showing that the shift in migration is due to phosphorylation (Figure 8B right panel).

In the above experiments, we could not distinguish between whether failure of Pdd1p dephosphorylation directly blocked foci formation, or whether *∆LIA5* cells arrested at a stage of development prior to the loss of this modification. To assess whether Pdd1p phosphorylation may inhibit foci formation, we examined the state of Pdd1p in *∆DCL1* cells. Mutations in components of the RNAi pathway, e.g. *∆DCL1* or *∆TWI1*, which lead to failure in scnRNA-directed heterochromatin formation, assemble Pdd1p foci in developing macronuclei as soon as these nuclei appear in cells (Figure 8C). While it is not clear how these foci relate to normal DNA elimination foci as chromatin modifications on IESs are not established, their presence suggest that Pdd1p can be partitioned into sub-nuclear domains in the absence of small-RNA directed heterochromatin targeting. If phosphorylation prevents foci assembly, Pdd1p should remain unphosphorylated in *ΔDCL* mutants. As in ∆*LIA5* cells, Pdd1p levels do not decrease in late time points (15 h, Figure S1). Furthermore, examination of Pdd1p isolated from these mutants show that the Pdd1p phosphorylated isoforms accumulate and, as in ∆*LIA5* cells, remains modified throughout conjugation (Figure 8B). Thus, phosphorylation does not appear to be a physical barrier to Pdd1p aggregation.

### Induced DNA damage is sufficient to recover Pdd1 protein dephospohrylation and foci formation in ∆*LIA5* conjugants

**Figure 9:**
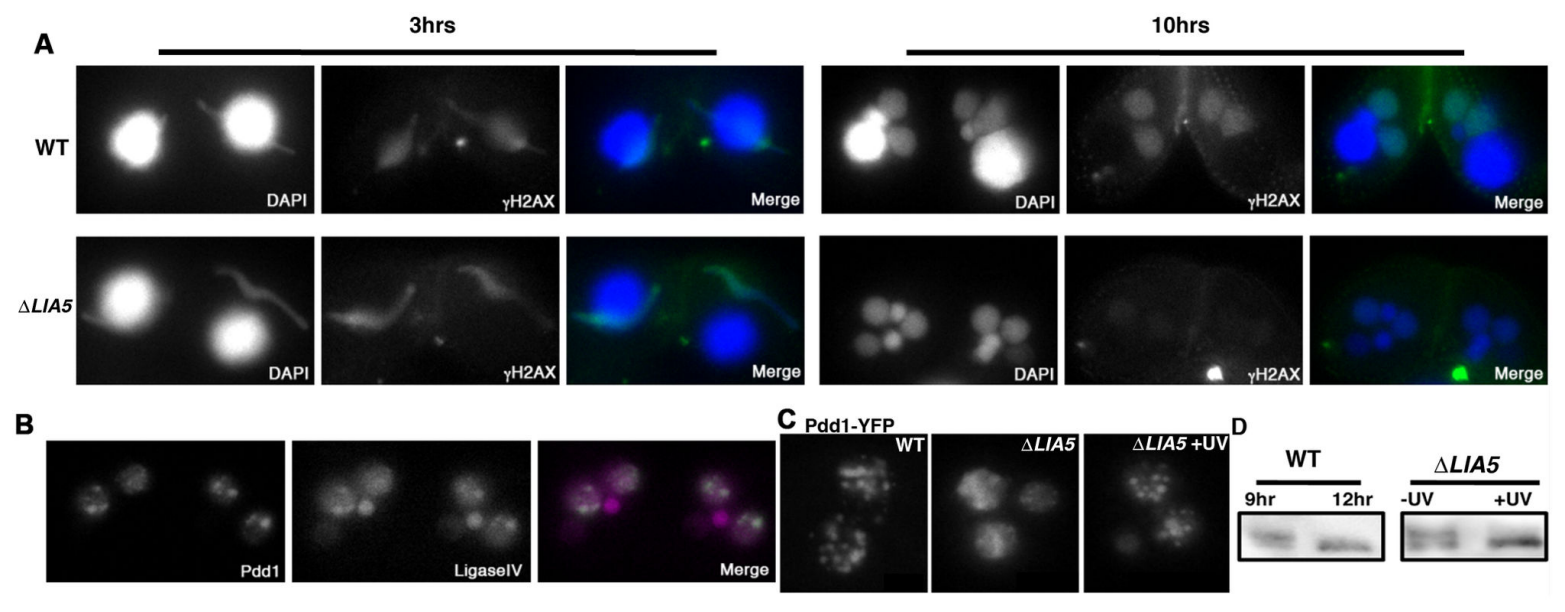
DNA damage rescues foci formation in *∆LIA5* **conjugants**. (A) Immunostaining of γH2AX in WT and *∆LIA5* during meiosis (3hrs) and macronuclear differentiation (10hrs) stages of conjugation. (B) Fluorescence images of Pdd1-CFP and LigaseIV-YFP co-localization after UV exposure. (C-D) UV induced DNA damage is sufficient to rescue Pdd1 localization and de-phosphorylation in Δ*LIA5*. (C) Pdd1-YFP localization in 14hrs conjugating WT, Δ*LIA5*, and Δ*LIA5* cells treated with 150mJ of UV. (D) anti-Pdd1 western blot analysis for WT mating at 9hrs and 12hrs. Irradiated (+UV) or control (-UV) *∆LIA5* mating cells at 12hrs.

The functions of DNA elimination foci are not known. Their assembly may bring together components of the DNA elimination machinery to facilitate the excision of the nearly 6000 dispersed IESs, or alternatively, they may form after excision to sequester excised IESs and/or aid in repair of the programmed DNA double strand breaks. The previous observation that foci do not form upon knockdown of the domesticated transposase, *TPB2*, suggests that excision may be a prerequisite to the formation of these sub-nuclear structures [32]. Our analysis of *∆LIA5* cells also suggests that IES excision may lead to foci formation as these mutants fail to initiate breaks (Figures 3 and 4) and Pdd1p remains dispersed (Figure 7). If indeed ∆*LIA5* cells fail to make programmed DNA breaks at IES junctions, they should not accumulate γH2AX. This conserved marker of the DNA damage response has been shown to accumulate in micronuclei during meiosis, when recombination-associated DNA breakage occurs [44]. Both wt and *∆LIA5* strains contain γH2AX in the meiotic nuclei (Figure 9A, 3 hrs); however, after developing macronuclei emerge, γH2AX was detected only in wt cells and was largely absent from *∆LIA5* macronuclei (Figure 9A, 10 hrs). Thus *∆LIA5* cells do not initiate this cellular response to double strand breaks, likely due to absence of IES excision.

The behavior of Pdd1p and γH2AX in ∆*LIA5* strains suggest that DNA elimination foci form as a response to programmed DNA breaks. If this is indeed the case, we reasoned that we could induce formation of Pdd1p foci by introducing DNA damage ectopically. Cells expressing Pdd1p-CFP were exposed to UV treatment at a stage where Pdd1p is normally dispersed in the developing macronucleus. We observed that Pdd1p assembled into foci in response to this treatment (Figure 9B). We recently found that the NHEJ protein, Ligase IV is concentrated into nuclear foci after UV treatment of growing cells, structures that we postulate are sites of DNA repair (Figure S2A). We therefore asked whether the UV-induced Pdd1p foci overlap with sites of Ligase IV accumulation. Ligase IV-YFP does not appear to be tightly associated with Pdd1p normally as this fusion protein is uniformly distributed in developing macronuclei throughout conjugation (Figure S2B). Upon UV treatment, Pdd1p and Ligase IV relocalize to some common sites in these nuclei (Figure 9B), which supports our assertion that Pdd1p is recruited to sites of DNA damage.

To further link DNA damage to DNA elimination foci formation, we treated *∆LIA5* cells with UV radiation. We found that introduction of ectopic DNA lesions rescued the foci formation phenotype in ∆*LIA5* conjugants (Figure 9B). To further examine whether this foci formation mimicked the programmed DNA elimination response, we examined the phosphorylation state of Pdd1p. Whereas phosphorylated Pdd1p accumulated in ∆*LIA5* mutants, UV treatment induced Pdd1p dephosphorylation (Figure 9C). Taken together, our data are consistent with the hypothesis that DNA elimination foci form as a consequence of a programmed DNA damage response.

## Discussion

*Tetrahymena thermophila* somatic nuclear differentiation requires genome-wide remodeling to generate the transcribed genome for the next generation. In this study, we found that *LIA5* encodes a protein critical for the chromosome breakage and DNA elimination events that fragment and streamline the genome for efficient gene expression. Lia5p acts after the establishment of RNAi-directed heterochromatin modifications in the zygotic genome (Figure 6), but prior to the elimination of the marked sequences (IESs). In conjugating *∆LIA5* cells, the unrearranged, micronuclear form of IESs accumulate (Figure 3), IESs circles are not detectable (Figure 4), and phosphorylation of H2AX (γH2AX) is severely diminished (Figure 9); all are observations that indicate failure to the initiate ds breaks at IES boundaries. Cells lacking *LIA5* display similar developmental phenotypes as *∆LIA1* cells and cells with knocked down expression of TPB2, which encodes the domesticated *piggyBac* transposase that performs IES excision [32,35]. In all three cases, heterochromatin modifications are established, but Pdd1p remains dispersed. Clearly, multiple proteins must cooperate to excise this newly established heterochromatin from the somatic genome after the specific sequences are identified by the Twi1p-scnRNA machinery.

### DNA elimination foci form in response to IES excision

The failure of *∆LIA5* cells to form DNA elimination foci led us to investigate what triggers the assembly of these structures. As Pdd1p, an Hp1-like chromodomain-containing protein, is a major component, it has been suggested that foci represent the mature form of newly established heterochromatin in the developing macronucleus (see 28,45). These foci grow larger in size and fewer in number, appearing to coalesce near the nuclear periphery as macronuclear differentiation proceeds [30]. In this model, heterochromatin is fully compartmentalized prior to its elimination, and Lia5p may act as an essential chromatin protein that participates in the sub-nuclear partitioning of IESs. We previously reported that GFP-Lia5p co-localizes with Pdd1p [30], but more comprehensive analysis here shows that Lia5p does not strongly associate with DNA elimination foci. Its localization surrounding Pdd1p structures remains consistent with its involvement in the nuclear reorganization of IESs into these structures. Nevertheless, the observation that these structures do not form in *∆LIA5* cells, or in other mutant lines that fail to excise IESs, is supportive of the equally likely possibility that foci normally develop in response to DNA breaks introduced upon IES excision [32,35]. Our data are fully consistent with this second hypothesis as we could induce Pdd1p to form foci in the absence of *LIA5* by introducing ectopic DNA damage (Figure 9).

The redistribution of repair proteins into sub-nuclear foci is a dynamic process induced upon DNA damage [6-8]. DNA repair foci have not been described in *Tetrahymena* and, before this study, the relationship between DNA elimination structures and repair foci was largely unexplored. We showed here that the NHEJ protein, Ligase IV, is recruited to foci containing Pdd1p in UV-treated mating cells, indicating that *Tetrahymena* repair proteins are reorganized upon DNA damage. We also found that Pdd1p is dephosphorylated in response to UV-induced DNA damage. These UV-induced events mimic what occurs normally upon IES excision in wild-type cells when DNA elimination foci form and Pdd1p is dephosphorylated, coincident with the introduction of programmed ds breaks. These data strongly suggest that the organization of IES heterochromatin into sub-nuclear structures is triggered by a DNA damage response and implicate Pdd1 as a target of this response. Hp1 is known to respond to DNA damage (reviewed in 10-12). Our findings show that heterochromatin proteins are involved in the repair of DNA damage in ciliates and raise the possibility that such roles are ancient and potentially evolutionarily conserved.

Host DNA repair processes have been shown to participate in ciliate programmed DNA rearrangements. The major NHEJ components Ligase IV and XRCC4 are required for DNA rearrangement that occur during *Paramecium* macronuclear differentiation [34], and the Tetrahymena Ku80 protein (Tku80p) was recently shown to play an essential role in *Tetrahymena* development as well [33]. Cells lacking Tku80 (*∆TKU80*) still excise IESs, but are unable to rejoin the resulting ds breaks. The unrepaired chromosomes are eventually degraded leaving developing macronuclei devoid of detectable DNA. This phenotype was first described for strains lacking *DIE5* in their micronuclei [40]. The observation that loss of late (zygotic) *DIE5* expression alone was sufficient to block completion of DNA rearrangement, which in turn led to wholesale degradation of DNA in developing macronuclei, suggested that IES excision occurred, but rejoining did not. We provide additional data (Figure 3B), that *∆DIE5* cells do not accumulate unexcised IESs during development. These *DIE5* mutants still formed Pdd1p foci, which is consistent with our assertion that foci form in response to DNA breaks at IESs.

Even though both *∆DIE5* and *∆TKU80* excise IESs then degrade all their macronuclear DNA, they differ in their generation of Pdd1p foci; *∆DIE5* cells form foci, *∆TKU80* cells do not. We interpret this to mean that that ability to respond to ds breaks at IESs is still intact in Δ*DIE5* strains, but not in Δ*TKU80* cells. We propose that Tku80 acts as a DNA damage sensor, without which, the cuts made by the Tpb2p transposase at IES boundaries go unrecognized, resulting in the failure to signal the formation of DNA elimination foci. In wild-type cells, Tku80p does not co-localize with Pdd1p, which we think further indicates that Tku80p’s main roles are to: sense breaks that occur at IESs, initiate a damage response that signals the formation of Pdd1p foci, and protect the free ends of macronucleus-destined sequences until rejoined. In this model, Die5p acts downstream of the Tku80p damage signal, assisting in the repair of new macronuclear genome after IES excision.

Even though our data indicated that DNA elimination structures form in response to DNA damage, several observations suggest to us that they may not be analogous to DNA repair foci in other eukaryotes. Pdd1p and γH2AX do not co-localize. Tku80p is not observed within DNA elimination structures, even though it is required to rejoin the macronucleus-destined DNA flanking IESs after their excision. This suggests that repair of the developing somatic genome occurs outside of foci. We believe these data indicate that formation of DNA elimination foci is more likely the response of heterochromatin to DNA damage. The repeat-rich DNA found in heterochromatin can be challenging to repair. The homologous recombination machinery may have difficulty distinguishing between an undamaged sister chromatid and other nearby homologous sequences when selecting a repair template, which could lead to aberrant genome rearrangements. In *Drosophila* , homologous recombination is repressed in heterochromatin until the free ends of damaged DNA are moved outside of the heterochromatin domain, where repair can occur without these complications [46]. Similar relocation of ds breaks outside of heterochromatin compartments has been observed in mammalian cells [47]. The formation of DNA elimination foci may represent a complementary phenomenon leading to the sequestration of the repeat rich IESs as heterochromatin away from repair proteins, promoting accurate joining of the retained genomic sequences.

### Transposons and the origin programmed DNA elimination

*LIA5* is a glutamine-rich, zinc finger protein that shares structural similarity with the IS4 family of transposases [36]. The *LIA5* coding region contains both DDE_Tnp_1_7 and Tnp_zf-ribbon_2 domains, a protein architecture that is shared by many transposon-derived proteins, including the *piggyBac* transposase. Therefore, like *TPB2* and *PGM*, which encode the IES excisases in *Tetrahymena* and *Paramecium* , respectively, *LIA5* is likely derived from a transposon, fixed in the genome through a molecular domestication event [31,32]. *Tetrahymena* cells employ the Lia5 protein to assist in the removal (i.e. enforce the silencing) of transposons and their remnants, the IESs. Domesticated transposases have important roles in essential cellular functions of diverse eukaryotic organisms (reviewed in 48). Among the most well known examples are the RAG1/2 recombinase and CENPB centromeric proteins of mammals and the *FAR1* and *FHY3* transcriptional regulators of plants. Analyses of genome sequences are revealing the prevalence with which transposons contribute to eukaryotic proteomes. Initial estimates showed that ~4% of human protein coding genes are or contain transposon-derived sequences [49,50]. Lia5p’s essential role in programmed DNA rearrangement suggests that multiple transposon proteins may have been co-opted in ciliates to defend the genome against these evading elements.

Although Lia5 has structural similarity to transposases, its putative DDD/E catalytic residues are not obviously conserved. Attempts to detect cleavage activity of Lia5p were unsuccessful (A. Vogt and K. Mochizuki, personal communication). Thus, despite its likely transposon origin, it is unlikely to directly cleave IES DNA, which is the function of Tpb2p [32]. The conserved zinc finger in Lia5 is likely critical for its function, possibly guiding its association with DNA or chromatin. Although the biochemical function of Lia5p remains to be determined, the lack of DNA elimination foci and γH2AX staining in its absence suggests it may be involved in recruiting TPB2, either directly or indirectly, to initiate DNA cleavage. Perhaps Lia5 acts to direct chromatin remodeling to promote access of IESs to the excision machinery, a step that can be partially bypassed by inducing similar remodeling through a DNA damage response. Further studies to elucidate Lia5p function and the origins of the *LIA5* gene should provide important insight into the regulation of *Tetrahymena* DNA rearrangement. In the ciliate *Oxytricha*, the excisase of its IESs may still be encoded by an active family of transposons [51,52]. Clearly, transposons continue to be involved in the evolution of novel processes that regulate eukaryotic genomes.

## Materials and Methods

### Cell lines and culture

*Tetrahymena thermophila* cells were grown in liquid culture at 30°C according to standard methods [53]. Wt strains (B2086, CU427, CU428) and the micronucleus-defective ‘star’ strains (B*VI, B*VII) were originally obtained from Peter Burns (Cornell University, Ithaca, NY) and are available from the Tetrahymena Stock Center (http://tetrahymena.vet.cornell.edu/). These strains or their progeny were transformed with constructs to create knockout strains or cell lines expressing epitope-tagged proteins. *∆*Dcl1 and ΔPdd1 strains were described [25,54]. Cells were made competent to mate by overnight starvation (>6 hours) in 10 mM Tris-HCl (pH 7.4), and conjugation was induced by mixing starved cultures of mating compatible strains at equal cell densities (~2.5x10^5^ cells/ml).

### RT-PCR

cDNA was synthesized from 4 µg of total RNA isolated at different stages of conjugation with SuperScript II reverse transcriptase (Invitrogen) [25,55]. Oligonucleotide primers designed to flank the 6^th^ intron (Lia5rtFw 5’-ttctctaggctaagcaccctaaaa-3’ Lia5rtRv 5’-tccattgtacccattgttcatt-3’). used to monitor *LIA5* expression by PCR.

### Generation of Lia5 knockout and expression strains

A *LIA5* knockout construct pLia5KO was generated using a Multisite Gateway Cloning kit (Invitrogen, Life Technologies, NY) as previously described [40,54]. DNA corresponding to regions upstream and downstream of the *LIA5* coding sequence was amplified from CU427 genomic DNA using the following primer pairs:

LIA5upFw 5’- GGGGACAACTTTGTATAGAAAAGTTggtacctacaaggacaatggcaccaa-3’ LIA5upRv 5’ GGGGACTGCTTTTTTGTACAAACTTGtggctaaaatttctgcagtcg-3’ and LIA5downFw 5’- GGGGACAGCTTTCTTGTACAAAGTGgccaatagataaaatggcacct-3’

LIA5downRv 5’- GGGGACAACTTTGTATAATAAAGTTggtacctcatttccgaaaaatatcat-3’, respectively (Uppercase letters are att sequences added to facilitate Gateway recombination). The PCR products were used in BP recombination reactions with donor vectors. The resulting clones were combined with the pENTR-Neo3 selection cassette [54] in a multi-plasmid LR clonase reaction into pDEST-R4-R3 to create pLia5KO. This construct was linearized with Acc65I (site underlined in primer sequences) and introduced by biolistic transformation into strains CU428 and B2086 between 2.5 to 3.5 hrs of conjugation to obtain micronuclear transformants [56,57]. Transformants were selected by growth in 1x Spp medium contain 1 µg/ml CdCl2 and 80 µg/ml paromomycin sulfate (Sigma, St. Louis, MO). Confirmed heterozygous germ line transformants were mated with star strains B*VI or B*VII, inducing genomic exclusion to produce excongugants with homozygous micronuclei [58]. Strains homozygous for the knockout allele in their micronuclei were subsequently crossed to produce complete Lia5 knockouts (*∆LIA5*) missing all copies of the gene from both the micro- and macronucleus.

A hemagluttinin (HA)-tagged *LIA5* expression construct was created using a two step overlapping PCR strategy [35] to introduced the HA coding sequence immediately after *LIA5* start codon using primers HALIA5upFw 5’-CACCGGGCCCtagctggcattttcaataaataaa-3’ with HALIA5upRv 5’-taatcaggaacatcataaggatacattttaaattaattagttttcaaaggggataacttc-3’ and HALIA5downFw 5’-ccttatgatgttcctgattatgctgaattaggagaagcagatttacatacatcac-3’ with HALIA5downRv 5- CTCGAGaaaatgtattagcagctttaaatgtc-3’. The HA coding sequence is italicized and introduced restriction enzyme sites (ApaI, XhoI) are underlined. Primers LIA5dsFw 5’- GGATCCtgatatttttcggaaatgagga-3’ and LIA5dsRev 5’-CCGCGGagcaagcaaaggcgaaaata-3’ were used to clone the *LIA5* downstream genomic sequence (BamHI and SacII sites are underlined). Amplified PCR products were inserted into p4T2 vector containing the histone H4 promoter driven NEO cassette [59] to create the p4T2-*HALIA5* knock-in construct, which was linearized with ApaI and SacII and introduced into the macronucleus of starved cells by biolistic transformation. Phenotypic assortment was achieved by passage of transformants in media with increasing concentrations of paramomycin sulfate until complete replacement of the *LIA5* locus with the *HA-LIA5* allele was achieved.

### Co-Immunoprecipitation and Western Blot analysis

Co-Immunoprecipitation of Lia5p with Pdd1p was performed as previously described [35] using the *HA-LIA5* strains. For western blot analysis, immunoprecipitated samples, or total protein (isolated from ~1x10^6^ cells at different stages of conjugation) were boiled with 1X Laemmli Sample Buffer. Protein samples were separated with 4% stacking, 9% resolving polyacrylamide gel elecrophoresis, transferred onto nitrocellulose membranes and detected with primary antibodies: polyclonal anti-Pdd1 (Abcam ab5338, 1:1000), anti-Pdd3 (Abcam ab5340, 1:1000), anti-HA (Covance PRB-101P, 1:500), and anti-trimethyl-Histone H3 (Lys4) (EMD Millipore 07-473, 1:5000) followed detection with by HRP–conjugated, goat anti-rabbit IgG as a secondary. Blots were then overlaid with Pierce supersignal west duro chemiluminesce substrate and imaged using a Fuji imager.

### IES excision analysis

Single cells from mating pairs were isolated and lysed for PCR as previously described [23,38]. The M IES was analyzed with two successive rounds of PCR with following nested primers: Round I primers: MIFw 5'-AGCTTAAACAAATGCCATATTGAG-3' MIRv 5’-AAGGGGGGTGGGGAGGGAGAAGGA-3’ Round II primers: MIIFw 5’-TACGATAGATCGACTGACGG-3’ MIIRv 5'-GTGGGGAGGGAGAAGGATTCAAC-3'. Analysis of other IESs was performed by PCR as described [19] except that whole cell genomic DNA isolated from mating populations between 18 and 30 hours, as indicated after cells were mixed to initiate pairing. Detection of excised IES circles was performed by using nested PCR on whole cell DNA isolated between 12 and 18 hours [33,41]. The following oligonucleotide primers were used: M IES Round 1 primers: M_circle1_RV, 5'-CCTTATTAAGTGATCTAA AGA CCCAAG-3’ and M_circle0.9_FW1, 5’-GAAACCCATCCCCCTTTTT-3’; M IES Round 2 primers: M_circle2_RV, 5-AACTTATTGAAATTCGGCTAACATTATG-3’ and M_circle0.9_FW2, 5’-TTGTCTTGAATGTTTACAAAAATGTG-3’; R IES Round 1 primers: R_circle1_FW2, 5’-TTTTTCTTGTCTTACTTCAAAC and R_circle1_RV,5’-TGAGTATCAAAT CTTATTTTAATT-3’; R IES Round 2 primers: R_circle2_FW, 5’-TTTAATTAGTCAGGTTATAGG-3’ and R_circle2_RV, 5’-CTTAATTCACGT AATCAAGGAC. Genomic DNA or Round 1 products were amplified by using Taq DNA polymerase for 25 cycles of amplification each round. PCR conditions: Round 1 for each IES and Round 2 for the M IES: 94°C for 30 seconds, 52°C for 30 seconds, 72°C for 1 minute. Round 2 for the R IES: 94°C for 30 seconds, 50°C for 30 seconds, 72°C for 30 seconds. One third of each reaction was analyzed on 1.6% agarose gels.

### Northern and Southern blot analyses

Small RNA and Chromosome breakage analysis were performed as previously described [25,40]. The *LIA5* locus probe was the 850bp fragment isolated from BglII/BamHI digest of the *HA-LIA5* construct (see Figure 2).

### Indirect immunoflourescence

Cells were fixed by adding 10ml of fixative (2 parts satuarated mercuric chloride plus 1 part 95% Ethanol) to 3mls of cells in 10mM Tris. After incubating for 5 min at room temperature, fixative was removed. The cells were washed once with 6mls of 100% methanol and then resuspended in 1ml of methanol. For antibody staining, cells were dropped directly onto the slides, dried and rehydrated with 1xTBS. Rehydrated cells were blocked with 1% BSA, 0.01% Tween 20 in 1xTBS. Primary Antibodies: polyclonal anti-H3K9me2 (EMD Millipore 07-441, 1:500, Billerica, MA), monoclonal anti-H3K27me3 (Abcam ab6002, 1:500, Cambridge, MA), polyclonal anti-HA (Covance PRB-101P, 1:500, Princeton, NJ), polyclonal anti-H2AvD (Rockland PS137, 1:1000, Gilbertsville, PA). Secondary Antibodies: anti-Rabbit and anti-Mouse Alexa488 and Alexa594 (1:500, Invitrogen, Life Technologies, NY).

### Pdd1p phosphorylation analysis

To resolve the different phospho-isoforms of Pdd1p, total protein were isolated from ~1x10^6^ cells at different stages of conjugation by boiling cells with 1X Laemmli Sample Buffer. Protein samples were separated with 4% stacking, 9% resolving polyacrylamide gel electrophoresis, transferred onto nitrocellulose membrane and blotted with polyclonal antiPdd1 antibody (Abcam 5338, 1:1000, Cambridge, MA). Alkaline phosphatase treatment was performed as previously described [42].

### UV irradiation

Irradiation was performed with GS Gene Linker^TM^ UV Chamber (Bio-Rad, Hercules, CA). Conjugating cells in 3 to 7mls of 10mM Tris-HCl (pH 7.4) were exposed to 100 to 150mJ of UV-C (254nm). Cells were then covered in aluminum foil to prevent photolyase repair and allowed to recover at 30°C for at least 6 hours before harvesting for assays.

## Supporting Information

Figure S1 **Pdd1p and Pdd3p stably accumulates in *ΔLIA5* cells.** Whole cell extracts isolated from mating populations of wild-type (wt CU428 crossed to B2086) were fractionated on 10% SDS-polyacrylamide gels and transferred to nitrocellulose. Pdd1p and Pdd3p were detected with specific antibodies (Abcam ab5338- 1:1000 and ab5340 -1:1000, respectively). Extracts from approximately 500 mating pairs were loaded in each lane. Equivalent loading was assessed by detection of Acetylated alpha tubulin (Sigma Aldrich T6793, 1:2000). Accumulation of Histone H3 K4me3 was also monitored as a measure of establishment of active chromatin in developing macronuclei (EMD Millipore 07-473, 1:5000). The position of migration of protein size standards is indicated on the left (Fermentas page-ruler prestained protein ladder). Bands detected by specific antisera are indicated on the right. The bracketed, lower Pdd1p band likely represents degradation products due to over-accumulation. Whereas in wt cells, Pdd1p and Pdd3 accumulate to maximum levels by 9h of conjugation before their levels steadily decrease, in *ΔLIA5* and *ΔDCL1* cells, both Pdd1p and Pdd3p accumulate by 9hrs and remain stable until at least 15 h. Pdd3 maximal levels are lower in *ΔDCL1* cells than in wt or *ΔLIA5* cells, possibly reflecting the decreased amount of H3K27me3 in these mutants.
(EPS)

Figure S2 **Ligase IV localizes to foci upon UV treatment but not during nuclear development.** (A) Tetrahymena transformants expressing a Ligase IV (TTHERM_00387050)-YFP fusion were exposed to 100 mJoules of 254 nm ultraviolet light using a BIORAD GS Gene-Linker UV Chamber. Cells were first washed out of growth medium and resuspended in 10 mM Tris (pH7.4). 1.5 mls of cells were exposed then returned to growth medium and allowed to recover in the dark. Aliquots of cells were examine by fluorescence microscopy 2, 4 and 6 hours after treatment. After 6 hours (as shown), a significant number of cells contained large nuclear foci and fiber-like structures throughout the nucleus. (B) Cells expressing Ligase IV-YFP were crossed with CU428 and allowed to complete conjugation. Fluorescence was visualized by immobilizing live cells in 2% methylcellulose and imaged on a Nikon E600 microscope. Mating pairs 8 hours into conjugation and exconjugants ~14 hours after pairing are shown. DIC imaging is shown adjacent to the fluorescence of the YFP fusion.
(EPS)
